# P-mTOR Expression and Implication in Breast Carcinoma: A Systematic Review and Meta-Analysis

**DOI:** 10.1371/journal.pone.0170302

**Published:** 2017-01-23

**Authors:** Xian-Fei Ding, Li-Feng Li, Xue-Liang Zhou, Li-Na Guo, Meng-Meng Dou, Yan-Yan Chi, Shao-Xuan Wu, Ya-Na Zhang, Zheng-Zheng Shan, Yi-Jie Zhang, Feng Wang, Qing-Xia Fan, Jie Zhao, Tong-Wen Sun

**Affiliations:** 1 Department of General ICU, The First Affiliated Hospital of Zhengzhou University, Zhengzhou, Henan, China; 2 Department of Oncology, The First Affiliated Hospital of Zhengzhou University, Zhengzhou, Henan, China; 3 Department of Pharmacy, The First Affiliated Hospital of Zhengzhou University, Zhengzhou, Henan, China; 4 Department of Gerontology, The First Affiliated Hospital of Zhengzhou University, Zhengzhou, Henan, China; 5 Department of Integrated Medicine, The First Affiliated Hospital of Zhengzhou University, Zhengzhou, Henan, China; 6 Department of MRI, The First Affiliated Hospital of Zhengzhou University, Zhengzhou, Henan, China; Seoul National University College of Pharmacy, REPUBLIC OF KOREA

## Abstract

**Objective:**

Phosphorylated mammalian target of rapamycin (p-mTOR) is a promising prognostic marker in many types of cancer. However, its survival benefit in patients with breast carcinoma remains unknown. The aim of the present study was to assess the relationship between p-mTOR expression and prognosis in breast carcinoma based on a systematic review and meta-analysis.

**Materials and Methods:**

Electronic databases (including Pubmed, Embase, ISI web of science, and Cochrane Library) were searched up to November 24, 2015. The outcome measures were hazard ratios (HRs) with 95% confidence interval (CI) for the association between the prognosis of breast carcinoma patients and p-mTOR expression. Primary end points were disease-free survival (DFS), overall survival (OS), and recurrence-free survival (RFS). Statistical analysis was performed with STATA 12.0.

**Results:**

Nine cohort studies including 3051 patients met full eligibility criteria. The pooled HRs (95% CI) for OS, DFS, and RFS were 0.84 (0.27–2.63), 0.71 (0.40–1.23), and 0.48 (0.20–1.18), respectively.

**Conclusions:**

Our findings suggested that p-mTOR overexpression was not significantly related to prognosis in breast carcinoma regarding OS and disease recurrence. Prospective studies are warranted to examine the association between p-mTOR expression and survival outcomes in breast carcinoma.

## Introduction

Breast cancer is the leading cause of cancer-related death in women worldwide, with a mortality rate of 14% in female cancer patients [1]. A better understanding of the pathogenesis of breast carcinoma and advances in surgical, endocrinological, radiation, and chemical therapies dramatically improved the prognosis of patients with breast cancer; however, the 5 year survival rate remains low. New signaling pathways in breast cancer have been identified, and therapeutic modalities targeting these pathways may continue to improve the outcomes of cancer treatment [2].

Several new drugs have been developed to target specific signaling pathways in breast cancer, such as Herceptin, targeting the human epidermal growth factor receptor 2 (HER2) [3]. Despite promising results in clinical trials, drug resistance and genetic variations limit their further application [3]. Recent studies identified a potential signaling pathway named PI3K/AKT/mTOR, which plays an important role in survival, growth, proliferation, transformation, metabolism, and angiogenesis of tumor cells [4, 5]. PI3K and Akt can be phosphorylated in response to growth factors and nutrients, which activates mTOR, a highly conserved typical serine/threonine protein kinase [6, 7]. mTOR consists of two independent functional complexes, mTORC1 and mTORC2, which can be phosphorylated to p-mTOR. mTORCs directly phosphorylate and activate ribosome S6 kinase 1 (S6K1) and eukaryotic translation initiation factor 4E-binding protein 1 (4EBP1) [8, 9]. Their effect on activating 4EBP1 inhibits their capacity to repress the mRNA cap-binding protein eukaryotic initiation factor 4E (eIF4E) [10, 11]. Consequently, the main function of the mTORC-mediated signaling pathway is to regulate cell proliferation by activating ribosome biogenesis and protein synthesis [12, 13]. Abnormal activation of the mTOR signaling pathway is associated with the development of many types of cancer [14, 15] and is present in 70% of breast carcinomas [16–18]. Therefore, the PI3K/AKT/mTOR pathway plays a key role in the development of breast carcinoma.

The effect of dysregulation of the mTOR signaling pathway on the survival of breast cancer patients has been reported extensively in recent years. However, the role of mTOR expression in the prognosis of breast cancer remains controversial. Based on the contradictory results from different studies, the aim of this meta-analysis was to assess the prognostic value of p-mTOR expression in patients with breast cancer.

## Materials and Methods

### Search strategy

A literature search was performed using Pubmed, EMBASE, ISI Web of Science, and the Cochrane Library databases, covering all papers published up to November 2015. The search terms used in this meta-analysis met the following criteria: (1) Phosphorylated mammalian target of rapamycin or p-mTOR; (2) “Breast carcinoma” [Mesh] or “Breast Neoplasm” or “Breast Cancer” or “Breast carcinoma”; (3) prognosis or survive.

### Eligibility criteria

All languages were included; publications including only the abstract were excluded because of incomplete information. Titles and abstracts were examined first to eliminate unrelated studies, such as studies on cell lines or animals, reviews, and studies on other diseases. Then, all remaining articles were screened carefully for eligibility. Eligible articles were considered in this meta-analysis if they met the following criteria: (1) Definitive diagnosis of primary breast carcinoma; (2) Immunohistochemistry (IHC) or western blotting was used to measure the expression level of mTOR or p-mTOR; (3) Evaluation of studies addressing the correlation between mTOR or p-mTOR expression and breast carcinoma patients’ overall survival (OS), disease-free survival (DFS), and recurrence-free survival (RFS) among women; (4) Hazard ratio (HR) and 95% confidence interval (CI) were provided or could be calculated; (5) Included the latest and most complete study when involving more than one same cohort study of patients. Either abstracts or full texts were examined by two reviewers (Li-Feng Li and Xue-Liang Zhou) independently. Discrepancies were resolved by discussion with the third reviewer (Li-Na Guo).

### Data extraction

Eligible studies were evaluated independently by two researchers (Meng-Meng Dou and Xue-Liang Zhou), and the dispute was resolved by discussion with the third reviewer (Li-Feng Li). The extracted information was as follows: (1) first author, year of publication, nationality of patients, expression level of p-mTOR, number of p-mTOR-positive and -negative patients, histological type, clinical stage, and HR and 95% CI of OS, DFS, RFS. The quality of eligible studies was assessed using the Newcastle-Ottawa Scale (NOS).

### Statistical analysis

HR and 95% CI were used to assess the relationships between p-mTOR expression and DFS, OS, and RFS. Overexpression of p-mTOR was a risk factor for poor prognosis in breast cancer, especially when the HRs for DFS, OS, and RFS were > 1 and the 95% CI did not overlap with 1. Some studies introduced the HR and 95% CI directly, whereas, in most studies, software (Engauge Digitizer Version 4. 1) could be used to analyze Kaplan-Meier survival curves and extract the HR and 95% CI values. This method was first reported by Parmar [19]. In addition, the Q test and *I*^2^ test were used to measure heterogeneity, which was not considered significant at *P* > 0.05 or *I*^*2*^ < 50%. The pooled HR of each study was calculated using the fixed-effects model (Mantel-Haenszel), or alternatively the random-effects model (DerSimonian and Laird). Provided heterogeneity existed, subgroup analysis was performed to explore potential sources of heterogeneity. One-way sensitivity analysis was used to assess the stability of the results. Begg’s funnel plot [20] and Egger’s linear regression [21] were used to assess potential publication bias. All analyses were performed using Stata 12.0 statistical software (Stata Corp LP, College Station, TX, USA). Differences with *P* < 0.05 (two-sided) were considered statistically significant.

## Results

### Study characteristics of the included literature

**Fig 1:**
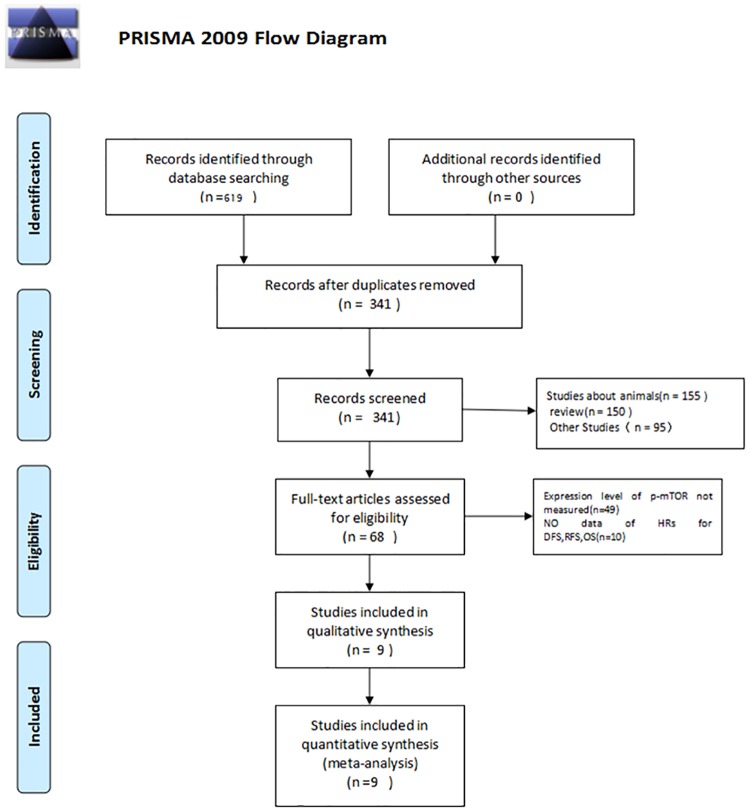
Flow diagram of study selection.

Nine articles involving 3051 patients [22–30] were included in the analysis after assessment of duplication (Fig 1). Three and five studies were analyzed by multivariate and univariate methods, respectively, and one study was analyzed using both methods. In the summary analysis, multivariate data were extracted when the results involved multivariate and univariate analyses, taking confounding factors into account. Eight studies provided integrated original information of the relationship between p-mTOR expression and clinical pathological parameters in breast carcinoma directly [22–25, 27–29]. One article assessed the prognostic value of p-mTOR (DFS) in breast carcinoma by the Kaplan-Meier method [26]. In all studies, IHC and paraffin-embedded specimens were used. The main characteristics of the nine cohort studies involved in our meta-analysis are shown in Table 1.

**Table 1:** Characteristics of studies on p-mTOR expression.

First author	Country	Year	No.of patients(mTOR high/low)	Age(years)	Pathological types(IDC/ILC/Other)	Stage(I/II/III/IV)	The median follow-up period(months)	HR(95% CI)of DFS	HR(95% CI)of OS	HR(95% CI)of RFS	IHC positive Cutoff value	Location of cell
Emiel A.M. J[19]	Netherlands	2007	125(81/44)	NA	NA	NA	134	NA	NA	0.6 (0.2–1.6)	≥10%	Cytoplasm
MA[20]	China	2015	285(206/79)	28–73	244/11/30	I, 65; II, 220	71.8	0.787(0.571–1.085)	NA	NA	>80%	Cytoplasm
Francisco B[21]	Canada	2015	331(145/186)	≤ 50,130; >50,201	228/18/85	NA	NA	0.40 (0.22–0.72)	0.26(0.11–0.61)	NA	NA	Cytoplasm
Georgios L[22]	Greece	2014	997(738/259)	NA	NA	NA	105	0.27(0.13–0.59)	NA	NA	NA	NA
Shikha B[23]	US	2006	138(33/105)	NA	138/0/0	NA	60	3.1 (1.27–7.56)	NA	NA	NA	Cytoplasm; Nuclear
Panagiotis B[24]	Greece	2010	192(131/61)	56 (25–87)	155/30/7	48/132/12/0	NA	NA	2.35(0.96–5.77)	NA	NA	Cytoplasm
Shir-Hwa U[25]	China	2012	172(124/48)	≤ 45, 65; >45,107	0/157/15	I-II, 125	68.84	NA	0.97 (0.51–1.84)	0.75 (0.43–1.3)	1–10%MSP,5–50%WP	Cytoplasm; Nuclear
Jungsuk A[26]	Korea	2010	530(207/136)	46.0 (26–85)	497/33/0	158/264/93/15	58.50	0.794(0.515–1.225)	NA	NA	≥10%SP,>50%WP	Cytoplasm; Nuclear
Karin B[27]	Netherlands	2014	421(89/332)	<65,210; ≥65,228	NA	I-II, 388; III-IV, 50	93.60	NA	NA	0.11 (0.02–0.55)	NA	Cytoplasm

Abbreviations: NA, information not available; IDC, invasive ductal carcinoma; ILC, invasive lobular carcinoma; SP, strong positive; MSP, moderately strong positive; WP, weak positive.

### Quality assessment

Quality assessment for eligible studies was performed using the NOS, which is recommended by the Cochrane Non-Randomized Studies Methods Working Group [31]. In this scale, studies are assessed based on three criteria, selection, comparability, and outcome. The quality of each study is graded with a maximum of eight stars. Grading was as follows: < five stars represented low quality and > six stars represented high quality. Quality assessment was performed by two investigators (Yan-Yan Chi and Shao-Xuan Wu), and any differences were resolved by Li-Na Guo. The NOS for quality assessment is shown in Table 2. All studies had adequate selection of patients, with true representation of the average breast carcinoma patients in the community. Long-term follow-up was sufficient to determine outcomes. For the other parts, scores were similar to each other.

**Table 2:** The Newcastle-Ottawa Scale (NOS) for assessing the quality of cohort studies.

RC studies	Selection			Comparability		Assessment of outcome			Total quality score
First author	Representativeness of mTOR positive arm	Selection of the comparative mTOR negative arm (s)	Ascertainment of mTOR positive regimen	Demonstration that outcome of interest was not present at start of study	Comparability between patients in different mTOR positive and negative arms-main factor: IHC positive Cut off value	Assessment of outcome with independency	Adequacy of Follow up length (to assess outcome)	Lost to follow up acceptable (less than 10% and reported)	HR(95% CI)of OS
Emiel A.M. J[19]	*	*		*	*	*	*		6
MA[20]	*	*	*	*	*	*	*		7
Francisco B[21]	*	*	*	*		*			5
Georgios L[22]	*	*		*		*	*		5
Shikha B[23]	*	*	*	*		*	*		6
Panagiotis B[24]	*	*	*	*		*			5
Shir-Hwa U[25]	*	*	*	*	*	*	*		7
Jungsuk A[26]	*	*	*	*	*	*	*		7
Karin B[27]	*	*		*		*	*		5

Abbreviations: RC, retrospective cohort study.

### Relationship between p-mTOR expression and DFS, OS, and RFS in breast carcinoma

**Fig 2:**
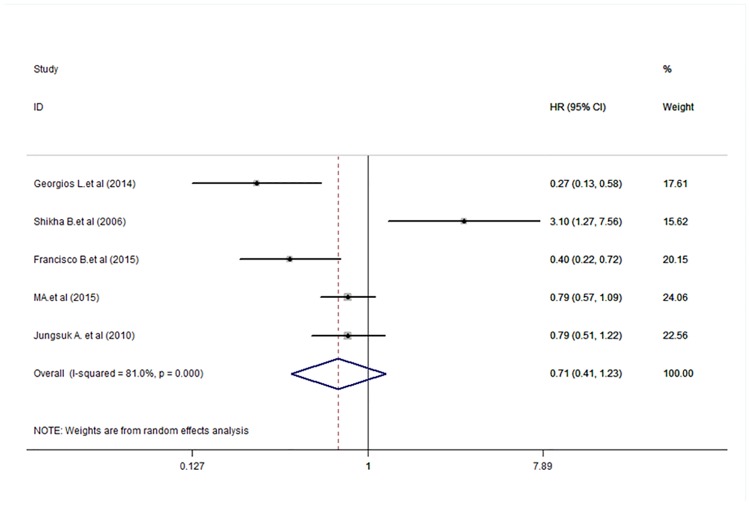
Meta-analysis of the overall pooled hazard ratios (HRs) of studies for the survival outcomes of breast cancer. Forest plot showing no statistical significance of the association between p-mTOR expression and disease-free survival of breast cancer patients from the random-effects model.

**Fig 3:**
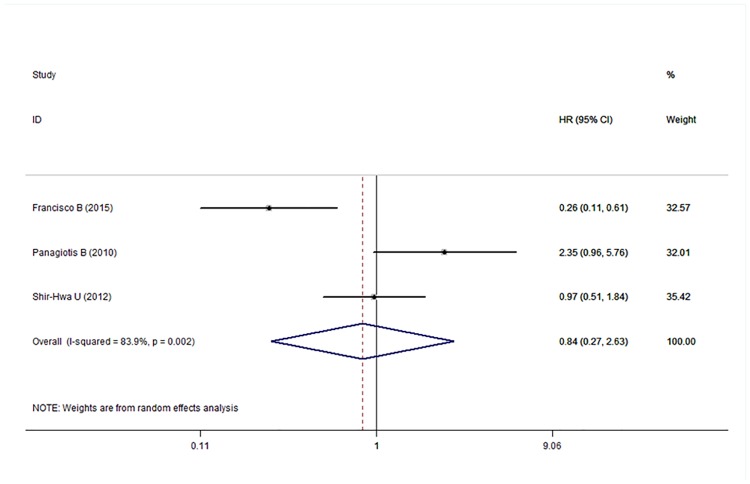
Meta-analysis of the overall pooled HR of studies for the survival outcomes of breast cancer. Forest plot showing no statistical significance of the association between p-mTOR expression and overall survival of breast cancer patients from the random-effects model.

**Fig 4:**
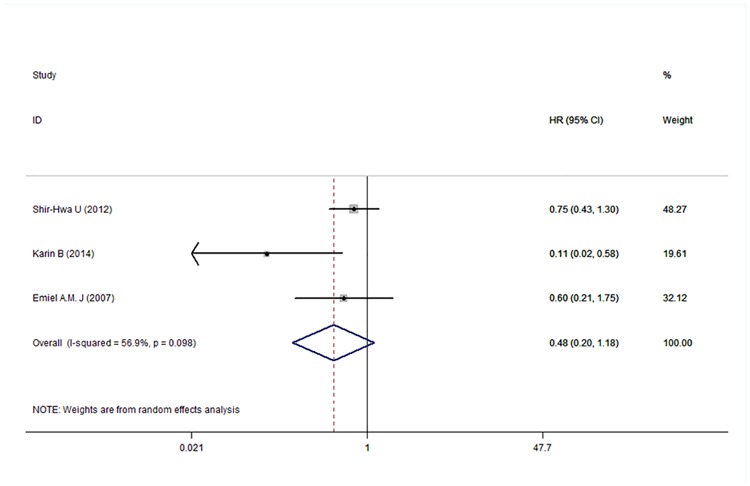
Meta-analysis of the overall pooled HR of studies for recurrence of breast cancer. Forest plot showing no statistical significance of association between p-mTOR expression and recurrence-free survival of breast cancer patients from the random-effects model.

Five [22–26, 29], three [24, 27, 28], and three [22, 28, 30]studies reported the HR and 95% CI for DFS, OS, and RFS, respectively. The results of the meta-analysis are shown in Figs 2–4. The pooled HRs for DFS, OS, and RFS were 0.71 (95% CI: 0.40–1.23), 0.84 (95% CI: 0.27–2.63), and 0.48 (95% CI: 0.20–1.18), respectively, which indicated no statistically significant relationship between p-mTOR expression and DFS, OS, and RFS in breast carcinoma patients. Therefore, we concluded that p-mTOR overexpression was not significantly related to the survival of breast carcinoma patients regarding disease recurrence and OS. A random-effects model was used to perform the meta-analysis because of mild or significant heterogeneity (respectively, *P* < 0.001, *I*^2^ = 81.0%; *P* = 0.002, *I*^2^ = 83.9%; and *P* = 0.098, *I*^2^ = 56.9%) among the studies.

### Subgroup analysis

**Fig 5:**
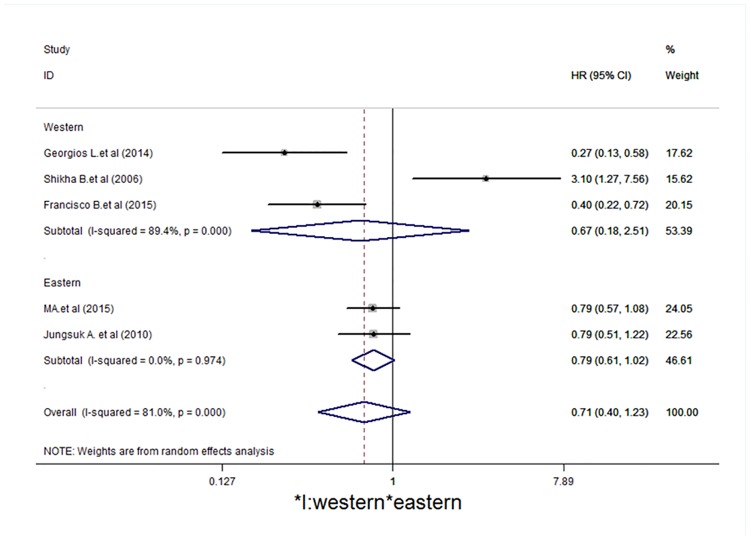
Subgroup analysis indicates that there is no statistical significance according to study region (Western and Eastern). Forest plot showing the association between p-mTOR expression and disease-free survival of breast cancer patients from the random-effects model.

**Fig 6:**
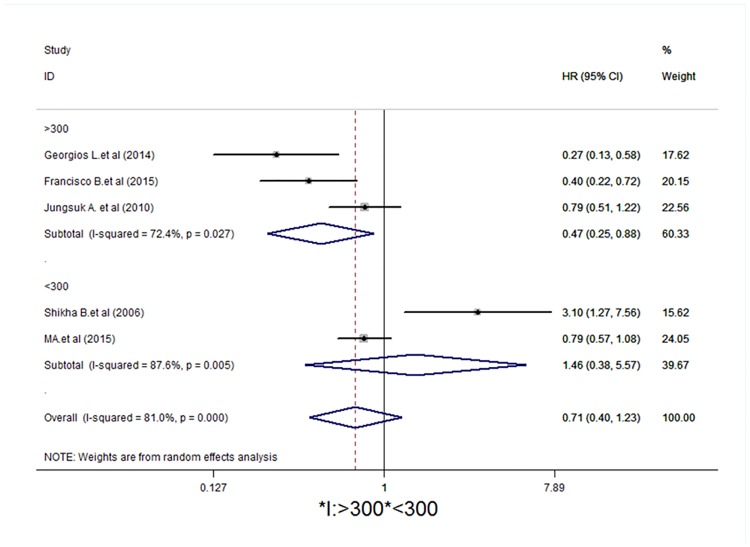
Subgroup analysis indicates statistical significance in the subgroup of > 300 patients. Forest plot showing the association between p-mTOR expression and disease-free survival of breast cancer patients from the random-effects model.

Studies were stratified to evaluate the HR of DFS according to p-mTOR expression by region (Western and Eastern) and the number of patients included in the study (> 300 patients and < 300 patients). The results of this subgroup analysis are shown in Figs 5 and 6. For the study region, studies from Eastern and Western countries showed no statistically significant differences (HR = 0.79, 95% CI = 0.61–1.02, *I*^*2*^ = 0.0%, *P* = 0.974; HR = 0.67, 95% CI = 0.16–2.51,*I*^*2*^ = 89.4%, *P* < 0.001, respectively). In the stratification by patients, the > 300 group showed statistical significance (HR = 0.47, 95% CI = 0.25–0.87, *I*^*2*^ = 72.4%, *P* = 0.027), whereas studies from the < 300 group had no statistical significance (HR = 1.48, 95% CI = 0.38–5.57,*I*^*2*^ = 81.0%, *P* < 0.001).

### Sensitivity analysis

**Fig 7:**
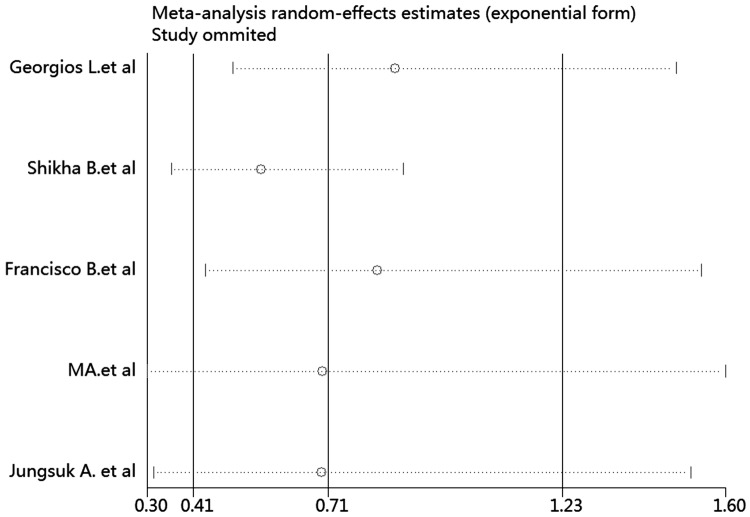
Sensitivity analysis showed that the studies were robust and reliable regarding p-mTOR expression and disease-free survival of breast cancer. The analysis was performed by excluding one study at a time and calculating the pooled estimate for the remaining studies.

Sensitivity analysis was performed to assess the influence of any one study on the pooled HRs and CIs by omitting one individual study at a time. Our findings showed that the results were robust and reliable (Fig 7) (data not shown).

### Publication bias

**Fig 8:**
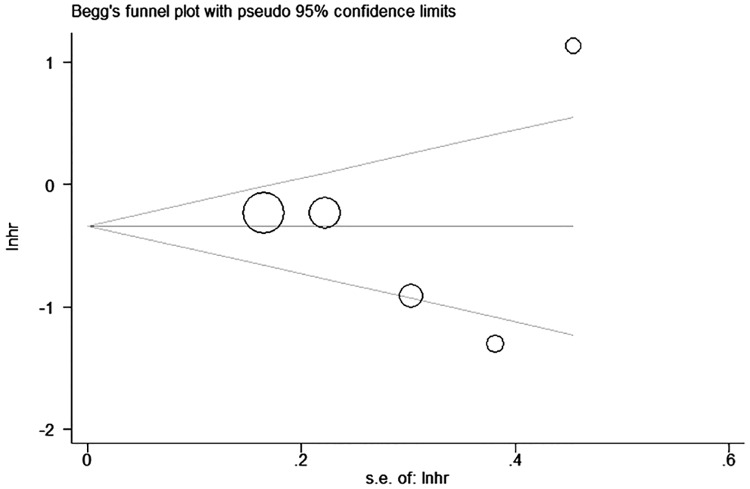
Begg’s funnel plot indicates no significant publication bias regarding p-mTOR expression and disease-free survival outcomes in breast cancer.

Begg’s funnel plots and Egger’s linear regression tests were performed to evaluate publication bias (Fig 8). No significant publication bias was found for the association between p-mTOR expression and DFS (Begg’s test, *P* = 0.806; Egger’s test, *P* = 0.954).

## Discussion

The present meta-analysis, which included nine articles and 3051 participants, assessed the significance of p-mTOR expression in breast cancer. The results showed that p-mTOR overexpression was not significantly associated with the survival of breast carcinoma patients regarding disease recurrence and OS. However, the heterogeneity of the Eastern subgroup was significantly reduced after stratification by region into Western and Eastern countries. Moreover, significant associations between p-mTOR expression and prognosis were identified in subgroups of > 300 patients.

mTOR is a 290 kDa molecule that functions as a serine-threonine kinase to regulate cell growth and metabolism through the mTOR signaling pathway [32]. Aberrant activation of the PI3K/Akt/mTOR signaling pathway is involved in oncogenesis and disease progression in breast carcinoma [26, 33, 34]. Uenget al.[28] reported that p-mTOR expression is an adverse prognostic indicator in early-stage (stage I and II) triple negative breast cancer, whereas it is not statistically significant in patients with stage III and IV. Bose et al.[26] showed that p-mTOR overexpression is associated with a 3-fold greater risk of disease recurrence in breast cancer. Zhou et al.[2] showed that p-mTOR is positively associated with HER-2 overexpression and correlated with poor DFS in patients with breast cancer. Bakarakos et al.[27] demonstrated that p-mTOR is positively associated with lymph node status and has a negative effect on survival outcome in invasive breast cancer. However, Annovazzi et al.[35] found no statistically significant association between p-mTOR expression and survival outcomes. A triple negative breast cancer study [28] showed that the majority of tumors (72.1%) are p-mTOR positive, whereas expression of p-mTOR is notcorrelated with tumor grade, lymph node status, and stage. Activation of the mTOR signaling pathway is associated with a more aggressive phenotype in both triple negative breast cancer and non-triple negative breast cancer. In short, the mechanism underlying the effect of the p-mTOR signaling pathway on breast cancer is complex, and numerous studies are necessary to elucidate it.

The prognostic significance of p-mTOR expression has been explored extensively in many types of cancer; however, the results remain controversial. Cai et al.[36]reported that overexpression of p-mTOR is associated with poor prognosis in early-stage breast carcinoma patients, with 5year survival rates of 32.7% and 56.4% for p-mTOR-positive and -negative patients, respectively. Survival analysis in a study by Leal[37] indicated that high p-mTOR expression is associated with poor prognosis in patients with advanced gallbladder adenocarcinoma. However, Valsamo et al.[38]showed that early-stage lung adenocarcinoma patients with high p-mTOR expression have a longer median OS than those with low expression. A meta-analysis performed by Lei Li et al.[39] reported no statistically significant association between p-mTOR expression and the prognosis of non-small cell lung cancer patients. Therefore, the clinical significance of p-mTOR expression in different cancers remains unclear.

mTOR inhibitors such as everolimus, which are targeted drugs in breast carcinoma, have been extensively tested in clinical trials for breast carcinoma. Everolimus in oral dosage form has been approved by the US Food and Drug Administration for the treatment of estrogen receptor-positive postmenopausal breast carcinoma patients [40]. In addition, a phase III randomized clinical trial conducted by Baselga et al.[41] showed that everolimus combined with exemestane improved progression-free survival (PFS) in patients with hormone receptor-positive advanced breast carcinoma that was previously treated, compared with the exemestane plus placebo arm. Similarly, a phase III, double-blind, randomized, international study (the BOLERO-2) [42] showed that the PFS of the everolimus plus exemestane arm was 8.5 months compared with 4.2 months in the placebo plus exemestane arm. A different study, BOLERO-3 [43], reported that the addition of everolimus to trastuzumab plus vinorelbine significantly prolonged PFS in patients with trastuzumab-resistant and taxane-pretreated, HER-2-positive, advanced breast carcinoma. These studies are inconsistent with the results of our meta-analysis, which could be attributed to the limited number of original articles included in the present meta-analysis.

The present meta-analysis has four advantages. First, it is the first meta-analysis assessing the clinical and prognostic role of p-mTOR expression in breast carcinoma. Second, the sensitivity analysis showed no significant difference when any one article of five was removed, which indicated that the association between clinicopathological parameters and DFS was relatively stable and credible. Third, no publication bias was detected. Finally, the original cases of the included studies had a good representation in breast carcinoma through strict inclusion and screening criteria, indicating that the results of the meta-analysis were reliable.

The present meta-analysis had several limitations. First, despite the fact that all studies met the inclusion criteria, the IHC cut-off points for the detection of positive or negative p-mTOR expression were not consistent. Second, some HR and 95% CI values obtained from Kaplan-Meier (K-M) curves were not precise. Finally, as the included studies did not provide complete information about patients, subgroup analysis according to pathological type and stage was not performed; the analysis was only stratified to evaluate the HR of DFS according to p-mTOR expression by region (Western and Eastern) and number of patients included the studies (> 300 patients and < 300 patients).

In conclusion, compared with low p-mTOR expression, p-mTOR overexpression was not significantly related to the prognosis of breast carcinoma patients regarding OS and disease recurrence. An updated meta-analysis including a larger number of original studies may provide further insight. Future studies should meet the following criteria: first, baseline characteristics should be balanced among groups, including country, race, pathological types, stage, tumor size, lymph node status, number of patients, median age, and follow-up period, with HR and 95% CI data for progression or death, adjuvant therapy, previous sensitivity to endocrine therapy, estrogen-receptor and ECOG performance status, and metastatic site. Second, adverse events and their grade, including anemia, hyperglycemia, fatigue, and pneumonitis, should be reported. Third, a sample number > 500 patients is needed.

## Supporting Information

S1 File PRISMA 2009 flow diagram. (DOC)

S2 File PRISMA 2009 checklist. (DOC)

## Abbreviations

4EBP1: transcription initiation factor 4E-binding protein 1
CI: confidence interval
DFS: disease-free survival
eIF4E: transcription initiation factor 4E
HR: hazard ratio
IHC: immunohistochemistry
OS: overall survival
p-mTOR: phosphorylated mammalian target of rapamycin
RFS: recurrence-free survival

## References

[1] Jemal A, Bray F, Center MM, Ferlay J, Ward E, Forman D. Global cancer statistics. CA: a cancer journal for clinicians. 2011;61(2):69–90. Epub 2011/02/08. 21296855 10.3322/caac.20107

[2] Zhou X, Tan M, Stone Hawthorne V, Klos KS, Lan KH, Yang Y, et al Activation of the Akt/mammalian target of rapamycin/4E-BP1 pathway by ErbB2 overexpression predicts tumor progression in breast cancers. Clin Cancer Res. 2004;10(20):6779–88. Epub 2004/10/27. 10.1158/1078-0432.CCR-04-0112 15501954

[3] Vogel CL, Cobleigh MA, Tripathy D, Gutheil JC, Harris LN, Fehrenbacher L, et al Efficacy and safety of trastuzumab as a single agent in first-line treatment of HER2-overexpressing metastatic breast cancer. Journal of clinical oncology: official journal of the American Society of Clinical Oncology. 2002;20(3):719–26. Epub 2002/02/01. 11821453 10.1200/JCO.2002.20.3.719

[4] Dobashi Y, Watanabe Y, Miwa C, Suzuki S, Koyama S. Mammalian target of rapamycin: a central node of complex signaling cascades. International journal of clinical and experimental pathology. 2011;4(5):476–95. Epub 2011/07/09. 21738819 PMC3127069

[5] Wullschleger S, Loewith R, Hall MN. TOR signaling in growth and metabolism. Cell. 2006;124(3):471–84. Epub 2006/02/14. 10.1016/j.cell.2006.01.016 16469695

[6] Hay N, Sonenberg N. Upstream and downstream of mTOR. Genes & development. 2004;18(16):1926–45. Epub 2004/08/18. 15314020 10.1101/gad.1212704

[7] Shaw RJ, Cantley LC. Ras, PI(3)K and mTOR signalling controls tumour cell growth. Nature. 2006;441(7092):424–30. Epub 2006/05/26. 10.1038/nature04869 16724053

[8] Holz MK. The role of S6K1 in ER-positive breast cancer. Cell cycle (Georgetown, Tex). 2012;11(17):3159–65. Epub 2012/08/17. 10.4161/cc.21194 PMC3466514 22895181

[9] Kenerson HL, Aicher LD, True LD, Yeung RS. Activated mammalian target of rapamycin pathway in the pathogenesis of tuberous sclerosis complex renal tumors. Cancer research. 2002;62(20):5645–50. Epub 2002/10/18. 12384518

[10] Asnaghi L, Bruno P, Priulla M, Nicolin A. mTOR: a protein kinase switching between life and death. Pharmacological research. 2004;50(6):545–9. Epub 2004/10/27. 10.1016/j.phrs.2004.03.007 15501691

[11] Mamane Y, Petroulakis E, LeBacquer O, Sonenberg N. mTOR, translation initiation and cancer. Oncogene. 2006;25(48):6416–22. Epub 2006/10/17. 10.1038/sj.onc.1209888 17041626

[12] Chung J, Grammer TC, Lemon KP, Kazlauskas A, Blenis J. PDGF- and insulin-dependent pp70S6k activation mediated by phosphatidylinositol-3-OH kinase. Nature. 1994;370(6484):71–5. Epub 1994/07/07. 10.1038/370071a0 8015612

[13] Hara K, Yonezawa K, Kozlowski MT, Sugimoto T, Andrabi K, Weng QP, et al Regulation of eIF-4E BP1 phosphorylation by mTOR. The Journal of biological chemistry. 1997;272(42):26457–63. Epub 1997/10/23. 9334222 10.1074/jbc.272.42.26457

[14] Yu G, Wang J, Chen Y, Wang X, Pan J, Li G, et al Overexpression of phosphorylated mammalian target of rapamycin predicts lymph node metastasis and prognosis of chinese patients with gastric cancer. Clin Cancer Res. 2009;15(5):1821–9. Epub 2009/02/19. 10.1158/1078-0432.CCR-08-2138 19223493

[15] Rolfo C, Giovannetti E, Hong DS, Bivona T, Raez LE, Bronte G, et al Novel therapeutic strategies for patients with NSCLC that do not respond to treatment with EGFR inhibitors. Cancer treatment reviews. 2014;40(8):990–1004. Epub 2014/06/24. 10.1016/j.ctrv.2014.05.009 24953979

[16] Lauring J, Park BH, Wolff AC. The phosphoinositide-3-kinase-Akt-mTOR pathway as a therapeutic target in breast cancer. Journal of the National Comprehensive Cancer Network: JNCCN. 2013;11(6):670–8. Epub 2013/06/08. 23744866 10.6004/jnccn.2013.0086 PMC4086482

[17] Stephens PJ, Tarpey PS, Davies H, Van Loo P, Greenman C, Wedge DC, et al The landscape of cancer genes and mutational processes in breast cancer. Nature. 2012;486(7403):400–4. Epub 2012/06/23. 10.1038/nature11017 22722201 PMC3428862

[18] Network CGA. Comprehensive molecular portraits of human breast tumours. Nature. 2012;490(7418):61–70. Epub 2012/09/25. 10.1038/nature11412 23000897 PMC3465532

[19] Parmar MK, Torri V, Stewart L. Extracting summary statistics to perform meta-analyses of the published literature for survival endpoints. Statistics in medicine. 1998;17(24):2815–34. Epub 1999/01/28. 9921604 10.1002/(sici)1097-0258(19981230)17:24<2815::aid-sim110>3.0.co;2-8

[20] Begg CB, Mazumdar M. Operating characteristics of a rank correlation test for publication bias. Biometrics. 1994;50(4):1088–101. Epub 1994/12/01. 7786990

[21] Egger M, Davey Smith G, Schneider M, Minder C. Bias in meta-analysis detected by a simple, graphical test. BMJ (Clinical research ed). 1997;315(7109):629–34. Epub 1997/10/06. 10.1136/bmj.315.7109.629 PMC2127453 9310563

[22] Janssen EA, Soiland H, Skaland I, Gudlaugson E, Kjellevold KH, Nysted A, et al Comparing the prognostic value of PTEN and Akt expression with the Mitotic Activity Index in adjuvant chemotherapy-treated node-negative breast cancer patients aged <55 years. Cellular oncology: the official journal of the International Society for Cellular Oncology. 2007;29(1):25–35. Epub 2007/04/13. 17429139 10.1155/2007/569246 PMC4618432

[23] Ma BL, Shan MH, Sun G, Ren GH, Dong C, Yao X, et al Immunohistochemical analysis of phosphorylated mammalian target of rapamycin and its downstream signaling components in invasive breast cancer. Mol Med Rep. 2015;12(4):5246–54. Epub 2015/07/08. 10.3892/mmr.2015.4037 26151180

[24] Beca F, Andre R, Martins DS, Bilhim T, Martins D, Schmitt F. p-mTOR expression is associated with better prognosis in luminal breast carcinoma. Journal of clinical pathology. 2014;67(11):961–7. Epub 2014/07/24. 10.1136/jclinpath-2014-202320 25053543

[25] Lazaridis G, Lambaki S, Karayannopoulou G, Eleftheraki AG, Papaspirou I, Bobos M, et al Prognostic and predictive value of p-Akt, EGFR, and p-mTOR in early breast cancer. Strahlentherapie und Onkologie: Organ der Deutschen Rontgengesellschaft [et al]. 2014;190(7):636–8, 40–5. Epub 2014/03/25. 10.1007/s00066-014-0620-6 24658605

[26] Bose S, Chandran S, Mirocha JM, Bose N. The Akt pathway in human breast cancer: a tissue-array-based analysis. Modern pathology: an official journal of the United States and Canadian Academy of Pathology, Inc. 2006;19(2):238–45. Epub 2005/12/13. 10.1038/modpathol.3800525 16341149

[27] Bakarakos P, Theohari I, Nomikos A, Mylona E, Papadimitriou C, Dimopoulos AM, et al Immunohistochemical study of PTEN and phosphorylated mTOR proteins in familial and sporadic invasive breast carcinomas. Histopathology. 2010;56(7):876–82. Epub 2010/07/20. 10.1111/j.1365-2559.2010.03570.x 20636791

[28] Ueng SH, Chen SC, Chang YS, Hsueh S, Lin YC, Chien HP, et al Phosphorylated mTOR expression correlates with poor outcome in early-stage triple negative breast carcinomas. International journal of clinical and experimental pathology. 2012;5(8):806–13. Epub 2012/10/17. 23071863 PMC3466984

[29] An J, Jeong H, Lee Y, Woo SU, Seo JH, Kim A. Phosphorylated Akt and Phosphorylated mTOR Expression in Breast Invasive Carcinomas: Analysis of 530 Cases. J Breast Cancer. 2010;13(4):337–48.

[30] Beelen K, Opdam M, Severson TM, Koornstra RH, Vincent AD, Wesseling J, et al Phosphorylated p-70S6K predicts tamoxifen resistance in postmenopausal breast cancer patients randomized between adjuvant tamoxifen versus no systemic treatment. Breast cancer research: BCR. 2014;16(1):R6 Epub 2014/01/23. 10.1186/bcr3598 24447434 PMC3979131

[31] Cota GF, de Sousa MR, Fereguetti TO, Rabello A. Efficacy of anti-leishmania therapy in visceral leishmaniasis among HIV infected patients: a systematic review with indirect comparison. PLoS neglected tropical diseases. 2013;7(5):e2195 Epub 2013/05/10. 10.1371/journal.pntd.0002195 23658850 PMC3642227

[32] Guertin DA, Sabatini DM. Defining the role of mTOR in cancer. Cancer cell. 2007;12(1):9–22. Epub 2007/07/07. 10.1016/j.ccr.2007.05.008 17613433

[33] Dillon RL, White DE, Muller WJ. The phosphatidyl inositol 3-kinase signaling network: implications for human breast cancer. Oncogene. 2007;26(9):1338–45. Epub 2007/02/27. 10.1038/sj.onc.1210202 17322919

[34] Heinonen H, Nieminen A, Saarela M, Kallioniemi A, Klefstrom J, Hautaniemi S, et al Deciphering downstream gene targets of PI3K/mTOR/p70S6K pathway in breast cancer. BMC genomics. 2008;9:348 Epub 2008/07/26. 10.1186/1471-2164-9-348 18652687 PMC2496917

[35] Annovazzi L, Mellai M, Caldera V, Valente G, Tessitore L, Schiffer D. mTOR, S6 and AKT expression in relation to proliferation and apoptosis/autophagy in glioma. Anticancer research. 2009;29(8):3087–94. Epub 2009/08/08. 19661320

[36] Cai W, Shi Y, Zhao Q. [Relationship between expression of mTOR and prognosis of early stage non-small cell lung cancer]. Zhonghua zhong liu za zhi [Chinese journal of oncology]. 2014;36(2):120–2. Epub 2014/05/07. 24796460

[37] Leal P, Garcia P, Sandoval A, Letelier P, Brebi P, Ili C, et al Immunohistochemical expression of phospho-mTOR is associated with poor prognosis in patients with gallbladder adenocarcinoma. Archives of pathology & laboratory medicine. 2013;137(4):552–7. Epub 2013/04/03. 23544944 10.5858/arpa.2012-0032-OA

[38] Anagnostou VK, Bepler G, Syrigos KN, Tanoue L, Gettinger S, Homer RJ, et al High expression of mammalian target of rapamycin is associated with better outcome for patients with early stage lung adenocarcinoma. Clin Cancer Res. 2009;15(12):4157–64. Epub 2009/06/11. 10.1158/1078-0432.CCR-09-0099 19509151

[39] Li L, Liu D, Qiu ZX, Zhao S, Zhang L, Li WM. The prognostic role of mTOR and p-mTOR for survival in non-small cell lung cancer: a systematic review and meta-analysis. PLoS One. 2015;10(2):e0116771 Epub 2015/02/14. 10.1371/journal.pone.0116771 25680114 PMC4332670

[40] Paplomata E, O'Regan R. The PI3K/AKT/mTOR pathway in breast cancer: targets, trials and biomarkers. Therapeutic advances in medical oncology. 2014;6(4):154–66. Epub 2014/07/25. 10.1177/1758834014530023 25057302 PMC4107712

[41] Baselga J, Campone M, Piccart M, Burris HA 3rd, Rugo HS, Sahmoud T, et al Everolimus in postmenopausal hormone-receptor-positive advanced breast cancer. N Engl J Med. 2012;366(6):520–9. Epub 2011/12/14. 10.1056/NEJMoa1109653 22149876 PMC5705195

[42] Ito Y, Masuda N, Iwata H, Mukai H, Horiguchi J, Tokuda Y, et al [Everolimus plus exemestane in postmenopausal patients with estrogen-receptor-positive advanced breast cancer—Japanese subgroup analysis of BOLERO -2]. Gan to kagaku ryoho Cancer & chemotherapy. 2015;42(1):67–75. Epub 2015/01/19. 25596682

[43] Andre F, O'Regan R, Ozguroglu M, Toi M, Xu B, Jerusalem G, et al Everolimus for women with trastuzumab-resistant, HER2-positive, advanced breast cancer (BOLERO-3): a randomised, double-blind, placebo-controlled phase 3 trial. The Lancet Oncology. 2014;15(6):580–91. Epub 2014/04/20. 10.1016/S1470-2045(14)70138-X 24742739

