# Supplementary material for: P-mTOR Expression and Implication in Breast Carcinoma: A Systematic Review and Meta-Analysis
Source: PLoS One. 2017 Jan 23;12(1):e0170302. doi: 10.1371/journal.pone.0170302 (PMC5256929; doi:10.1371/journal.pone.0170302)
Supplement: S1 File — (DOC) [file pone.0170302.s001.doc]

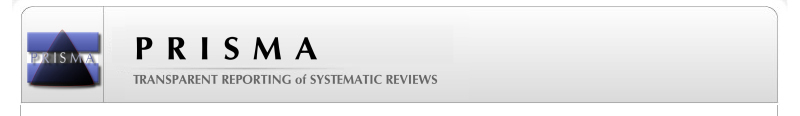
**PRISMA 2009 Flow Diagram**

**Screening**

**Included**

**Eligibility**

**Identification**

Records identified through database searching
(n =619 )

Additional records identified through other sources
(n = 0 )

Records after duplicates removed
(n = 341 )

Records screened
(n = 341)

Studies about animals(n = 155 )

review(n = 150 )

Other Studies（ n = 95）

Full-text articles assessed for eligibility
(n = 68 )

Expression level of p-mTOR not measured(n=49)

NO data of HRs for DFS,RFS,OS(n=10)

Studies included in qualitative synthesis
(n = 9 )

Studies included in quantitative synthesis (meta-analysis)
(n =9 )
